# A comprehensive meta-analysis on the efficacy of growth factor enriched cryo-agents in enhancing post-thaw quality of mammalian semen

**DOI:** 10.5713/ab.24.0898

**Published:** 2025-04-28

**Authors:** Suyatno Suyatno, Herdis Herdis, Tri Puji Priyatno, Mulyoto Pangestu, Santoso Santoso, Tatan Kostaman, Mohammad Firdaus Hudaya, Pangda Sopha Sushadi, Florentina Bety Indah Lupitasari, Anita Hafid, Zultinur Muttaqin, Ita Margaretha Nainggolan, Mohammad Miftakhus Sholikin, Pradita Iustitia Sitaresmi

**Affiliations:** 1 Research Center for Animal Husbandry, National Research and Innovation Agency, Cibinong Science Center, Bogor, Indonesia; 2 Education Program in Reproduction and Development, Department of Obstetrics and Gynecology, Monash Clinical School, Monash University, Clayton, Australia; 3 Research Center for Applied Zoology, National Research and Innovation Agency, Cibinong Science Center, Bogor, Indonesia; 4 Eijkman Research Center for Molecular Biology, National Research and Innovation Agency, Cibinong Science Center, Bogor, Indonesia

**Keywords:** Growth Factor, Insulin Growth Factor-1, Liposome, Mammalian Semen, Mesenchymal Stem Cells

## Abstract

**Objective:**

This study sought to evaluate the effect of growth factors (GFs) in semen extenders on the quality of post-thaw mammalian sperm using a meta-analysis approach. The main objective was to determine whether the addition of GFs could improve semen quality after cryopreservation.

**Methods:**

A meta-analysis of various *in vitro* experiments using mammalian semen was conducted. Data were collected from multiple studies assessing the effects of GFs on sperm motility, viability, acrosome integrity, DNA integrity, and other key semen quality metrics. The analysis included a range of mammalian species, incorporating specific GFs into semen extenders during cryopreservation. The evaluation of sperm quality was conducted using parameters including motility, viability, acrosome integrity, plasma membrane integrity (PMI), DNA integrity, hyper-osmotic swelling test (HOST), malondialdehid (MDA), and computer-assisted semen analysis (CASA). Statistical analyses, including standardized mean differences (SMD), were performed to compare the effects of GF additives with control treatments.

**Results:**

The addition of GFs into semen extenders significantly improved semen quality across various parameters, including motility, viability, acrosome integrity, and DNA integrity. The SMDs for motility, viability, acrosome integrity, PMI, DNA integrity, HOST, MDA, and CASA parameters were significantly higher in the GF-treated groups than those of the controls, with observed values of 2.56±0.303, 3.53±0.423, 1.22±0.351, 1.82±0.362, 8.73±2.514, 2.02±0.426, and 6.30±2.87, respectively. Notably, the addition of GFs maintained semen quality in most mammalian species (p<0.05, SMD>0.5), with the exception of boar semen.

**Conclusion:**

The present study demonstrated that the addition of GFs into semen extenders significantly enhances semen quality during cryopreservation across various mammalian species. This improvement is likely due to the antioxidants and repair factors found in the GFs. Each GF appeared to exert a distinct effect on sperm, subsequently enhancing sperm viability after thawing. The findings have important implications for improving reproductive technologies in mammalian species, particularly regarding cryopreservation and artificial insemination procedures.

## INTRODUCTION

Enhancing the efficiency of assisted reproductive technology involves the application of sperm freezing via cryopreservation, a highly effective method for the long-term preservation of genetic material [1]. Cryopreservation subjects cells to extremely low temperatures, halting metabolic activity to prolong viability [2]. This method also facilitates the transportation of sperm between distant locations [3]. However, despite its benefits, cryopreservation can lead to sperm quality deterioration after thawing. Mammalian spermatozoa are susceptible to cryo-injury and post-thaw injury, including ultrastructural damage, oxidative stress, and compromised motility and viability [4,5]. These damages arise from physical, chemical, and biochemical factors, as well as oxidative processes during semen extender administration and gradual chilling until thawing [6] and gradual chilling until thawing [7]. Oxidative stress, in particular, alters sperm membrane and DNA integrity, while ice crystal formation and chemical toxicity cause structural and functional damage. These injuries often lead to significant reductions (>50%) in sperm quality and function, especially during fertilization [8]. To mitigate these effects, various compounds, such as cryoprotective agents (e.g., antioxidants), anti-apoptotic agents, and growth factors (GFs), have been explored for their protective properties [9]. GFs are known to enhance metabolic processes, promote cell growth, and reduce oxidative stress. In sperm diluents, GFs help improve sperm kinematics, mitochondrial integrity, and cryo-tolerance [10]. These GFs could improve sperm kinematic parameters and mitochondrial membrane integrity, reduce lipid peroxidation, decrease apoptotic levels, enhance plasma membrane integrity (PMI), and improve cryo-tolerance in frozen sperm [11]. Various GF-containing derivatives, including liposomes [12], insulin growth factor-1 (IGF-1) [13], mesenchymal stem cells (MSCs) [14], exosomes, and micro-vesicles [15] have been utilized as adjuncts in cryo-agents to enhance the performance of post-thawed semen quality. This study aims to provide comprehensive data on the effectiveness of GF agents in frozen semen diluents by examining various semen quality parameters through a meta-analysis.

## MATERIALS AND METHODS

### Article search and selection protocol

**Figure 1:**
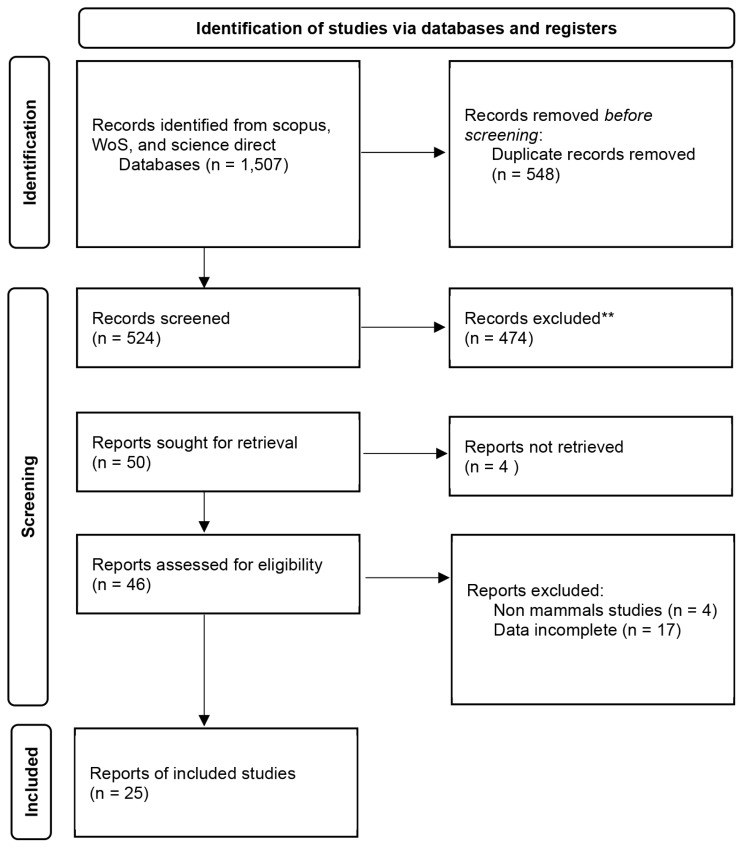
Illustrates the procedure of article selection and assessment in accordance with the PRISMA protocol. PRISMA, Preferred Reporting Items for Systematic Reviews.

A literature search was conducted using literature search engines, which were ISI Web of Knowledge (http://apps.isiknowledge.com), Scopus (http://www.scopus.com/), and Google Scholar (www.googlescholar.com), without any restriction on the period. MESH terms such as “stem cell,” “exosome,” “liposome,” “IGF-1,” “semen,” “cryopreservation,” “sperm,” and other relevant terms associated with *in vitro* studies were incorporated (Figure 1). In the initial phase, a search on Science Direct and Scopus yielded a total of 1,057 results using an algorithmic search approach for published articles (Figure 1). The inclusion criteria were implemented within a comprehensive framework covering population, intervention, comparators, outcomes, and study design. The primary interest was directed towards the effects of GFs on frozen mammalian semen. The focus was primarily placed on assessing motility, viability, and PMI sperm after thawing, but articles that were consistently composed in English and had been peer-reviewed before publication were also considered. Following these criteria, data were compiled and extracted from an initial dataset after a comprehensive review of 25 studies focusing on *in vitro* experiments. The guidelines set forth by the Preferred Reporting Items for Systematic Reviews (PRISMA) protocol were followed, and suitable articles were deliberately selected for analysis. The collection of published materials was expertly managed using the Mendeley references manager. This process involved documenting author names, publication years, study particulars (journal/article), types of GF agents, and the outcomes observed [16].

### Studies coding

A systematic selection process based on abstracts and full-text content was conducted following inclusion criteria, which identified 25 papers containing 16 sets of data (Table 1). Titles, abstracts, and full-text materials were assessed, considering information pertinent to variables such as liposome, exosome, MSCs, and IGF-1 levels on the effect of semen quality, mostly on sperm motility after thawing. The assembled database encompassed the inclusion levels of additive cryopreservation agent supplementation. Diverse mammals were employed in the study, including buffaloes, bulls, rams, bucks, canines, cats, humans, and stallions. This comprehensive research encompassed *in vitro* studies to explore various variables. In the in vitro component of the study, the focus was placed on assessing sperm quality. The mean values and standard deviations from all parameters used were collected and tabulated; also, the measurement units were homogenized for further data analysis. The main variables were discussed in depth, including semen motility, semen viability, intact acrosome, and PMI. Other parameters, such as the results of malondialdehid (MDA) and hyper-osmotic swelling test (HOST) and various sperm kinematic parameters, were explained briefly due to the difficulty of obtaining data in the tabulated articles. Some semen quality parameters were excluded due to the lack of data. Furthermore, these studies only explained that GF addition could maintain sperm quality post-thawing. However, none of the collected studies showed sperm capability to fertilize oocytes either *in vivo* or *in vitro*.

### Statistical analysis

Statistical analysis for this study was adapted from a previous study [16]. The effect size, represented as ‘Hedges’ d,’ was applied to quantify the parameter distance between conventional and organic products. This method was selected because of its ability to calculate the effect size regardless of heterogeneity in sample size, measurement units, and statistical test results. It was deemed suitable for estimating the effect of paired treatments [17]. The conventional group was pooled into a control group (C), and the GFs group was pooled into an experimental group (E).


(1) $$d=\frac{X^{E}-X^{c}}{S}J$$

Where *X^E^* was defined as the mean value from the experimental group and *X^C^* was defined the mean value of the control group. A positive effect size indicated that the parameter observed was greater in the organic group, and vice versa. J was defined as the correction factor for small sample size, calculated as:


(2) $$J=1-\frac{3}{\left(4\left(N^{C}+N^{C}-2\right)-1\right)}$$

and S was calculated as the pooled standard deviation, defined as:


(3) $$S=\sqrt{\frac{\left(N^{E}-1\right){\left(s^{E}\right)}^{2}+\left(N^{C}-1\right){\left(s^{C}\right)}^{2}}{\left(N^{E}+N^{C}-2\right)}}$$

Where N^E^ represented the sample size of the experimental group, N^C^ represented the sample size of the control group, s^E^ was the standard deviation of the experimental group, and s^C^ was the standard deviation of the control group. The variance of Hedges’ d (v_d_) was described as


(4) $$V_{d}=\frac{\left(N^{C}+N^{E}\right)}{\left(N^{C}N^{E}\right)}+\frac{d^{2}}{\left(2\left(N^{C}+N^{E}\right)\right)}$$

Cumulative effect size (d_++_) is formulated as


(5) $$d_{++}=\frac{\sum_{i=1}^{n}w_{i}d_{i}}{\sum_{i=1}^{n}w_{i}}$$

Where w_i_ was calculated as the inverse of the sampling variance: w_i_ = 1/v_d_. The precision of the effect size was described using 95% confidence interval (CI), i.e. d±(1.96×s_d_). All the equations above are derived from previous studies. The calculated effect size is statistically significant if the CI does not reach the null effect size [17].

The safety number (N_ft_) is calculated to recognize the publication bias caused by the insignificant studies not included in the analysis. N_ft_>5N+10 is considered to provide evidence of a robust meta-analysis model. N_ft_ was calculated using Rosenthal’s [16] method. with the last sample size from individual studies used as N. Cohen’s benchmarks were used to classify the magnitude of the effect size: 0.2 was considered small, 0.5 medium, and 0.8 large. A cumulative effect size calculation was conducted for various clusters, including variables like liposomes, MSCs, IGF-1, exosomes, and microvesicles (MVs), to assess the impact of moderator variables on the final effect size.

All effect size-related calculations were performed using MetaWin 2.0 software. A weighted paired-sample t-test was applied to the parameters, with the last sample size from individual studies used as the weighting factor. The mixed-effect model of analysis of variance (ANOVA) with Tukey’s test was applied to examine the influence of moderator variables on the parameters [16]. These tests were conducted using R program software.

## RESULTS

**Figure 2:**
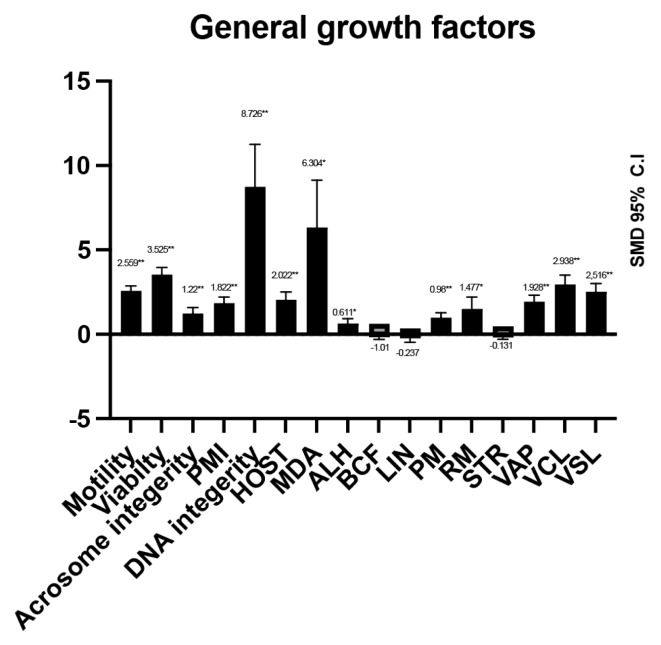
Sum mean different (SMD) of additive growth factor effect on post-thawing semen quality. Measurements and data collected are only data from post-thawing sperm quality. HOST, hyper-osmotic swelling test; MDA, malondialdehid; ALH, amplitude of lateral displacement; BCF, beat cross frequency; LIN, linearity; PM, progressive motility; RM, rapidity of movement; STR, straightness; VAP, velocity of average path; VCL, velocity of curvilinear line; VSL, velocity of straight line.

The investigation explored the influence of general GFs as cryopreservation agents. Compared to the control, the effect size (standardized mean differences, SMD) for sperm viability and motility was 3.53±0.43 and 2.56±0.303, respectively (Table 2). A positive correlation was found between higher GF concentrations and improved sperm viability and motility. Additionally, nearly all sperm quality indicators showed favourable outcomes. The best results were observed for DNA integrity and MDA values (8.73±2.51 and 6.30±2.82, respectively) (Figure 2). However, some computer-assisted semen analysis (CASA) parameters, including beat cross frequency (BCF; −0.11±0.22), linearity (LIN; 0.24±0.26), and straightness (STR; −0.13±0.18), showed negative SMD values, but these differences were not statistically significant compared to the control. Growth hormones exhibited varying effects on sperm motility across species such as humans, buffalo, bulls, dogs, mice, rams, and stallions, with a SMD of 2.56±0.303. In contrast, boars showed an SMD of −0.11±0.388, but suggesting no significant effect on sperm motility. Among the GFs, exosomes (SMD 4.78±1.02), IGF-1 (SMD 1.83±0.38), MVs (SMD 4.55±0.79), and MSCs (SMD 5.67±1.13) were positively associated with post-thaw-sperm motility. On the other hand, liposomes did not show a significant effect in sperm motility. These varying effects are clearly depicted in Figure 2.

**Figure 3:**
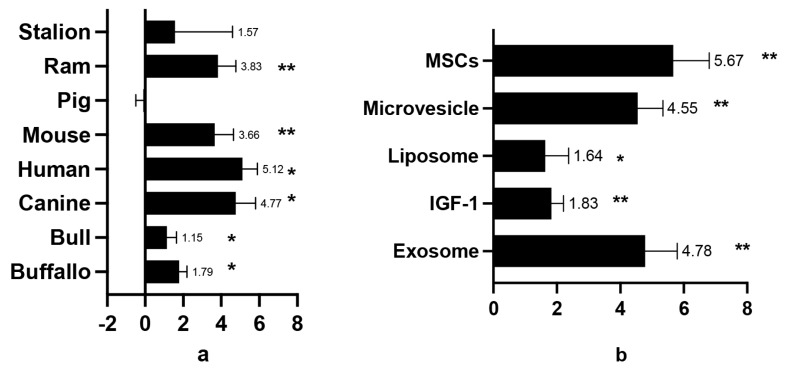
Sum mean different (SMD) of additive GFs effect on post-thawing semen motility in each species (a); SMD of each additive GFs in order to maintain sperm motility in post thawed semen (b). Measurements and data collected only data from post-thawing sperm quality. * means p<0.05, and ** mean p<0.01. MSCs, mesenchymal stem cells; IGF-1, insulin growth factor-1; GFs, growth factors.

**Figure 4:**
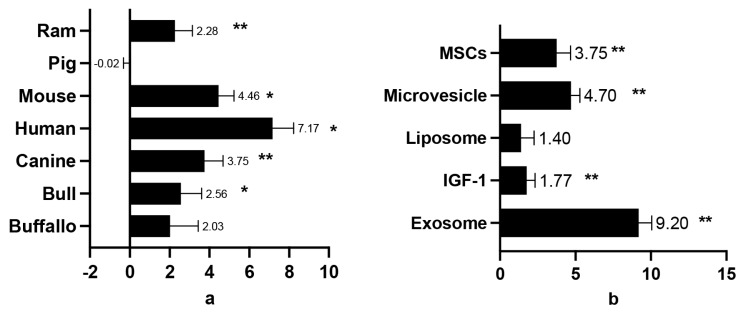
Sum mean different (SMD) of additive GFs effect on post-thawing semen viability in each species (a); SMD of each additive GFs in order to maintain sperm viability in post thawed semen (b). Measurements and data collected only data from post-thawing sperm quality. * means p<0.05, and ** mean p<0.01. MSCs, mesenchymal stem cells; IGF-1, insulin growth factor-1; GFs, growth factors.

**Figure 5:**
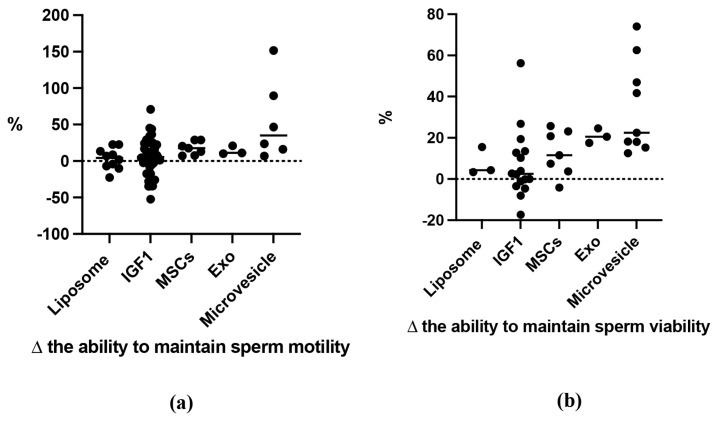
Percentage increase and decrease in the capability of growth factor in maintaining sperm motility (a) and viability (b) compared to control. IGF-1, insulin growth factor-1; MSCs, mesenchymal stem cells.

GFs supplementation, particularly in humans, was found to significantly improve sperm viability. Similar positive outcomes were observed in animal studies, including those on mouse, canine, bull, and ram sperm. However, GF supplementation did not yield statistically significant results for buffalo sperm. Notably, in boars, supplementation was associated with a decrease in sperm viability (Figure 3), indicating a detrimental effect occurred. Among the various GFs, exosomes exhibited the most pronounced positive effects on sperm viability, with SMD values ranging from 7.52 to 10.88. Micro-vesicles, MSCs, and IGF-1 also showed positive effects, but to a lesser extent. Micro-vesicles, exosomes, MSCs, and liposomes were ranked highest in their ability to maintain sperm viability, while IGF-1 demonstrated the lowest effectiveness (Figures 4, 5).

**Figure 6:**
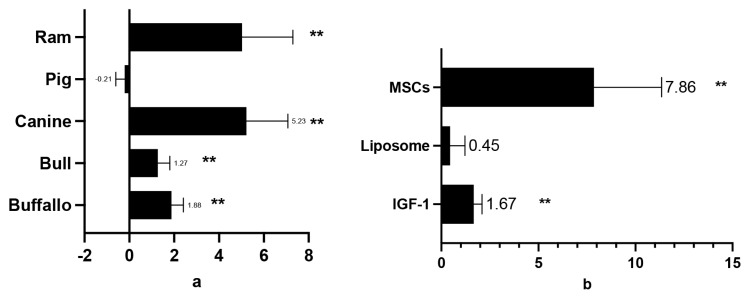
Sum mean different (SMD) of additive GFs effect on post-thawing semen PMI in each species (a); SMD of each additive GFs in order to maintain sperm PMI in post thawed semen (b). Measurements and data collected only data from post-thawing sperm quality. * means p<0.05, and ** mean p<0.01. MSCs, mesenchymal stem cells; IGF-1, insulin growth factor-1; GFs, growth factors; PMI, plasma membrane integrity.

**Figure 7:**
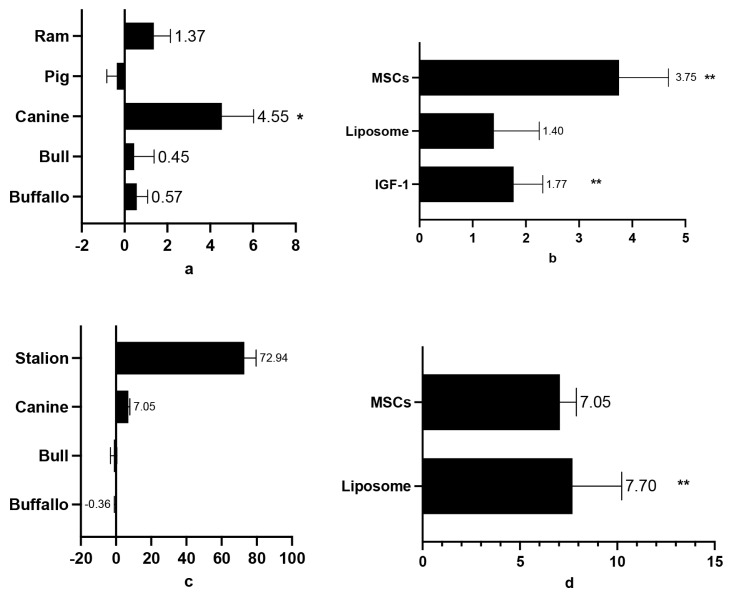
Sum mean different (SMD) of additive GFs effect on post-thawing semen acrosome integrity in each species (a); SMD of each additive GFs in order to maintain sperm acrosome integrity in post thawed semen (b). Sum mean different (SMD) of additive GFs effect on post-thawing semen DNA integrity in each species (c); SMD of each additive GFs in order to maintain sperm DNA integrity in post thawed semen (d). Measurements and data collected only data from post-thawing sperm quality. * means p<0.05, and ** mean p<0.01. MSCs, mesenchymal stem cells; IGF-1, insulin growth factor-1; GFs, growth factors.

**Figure 8:**
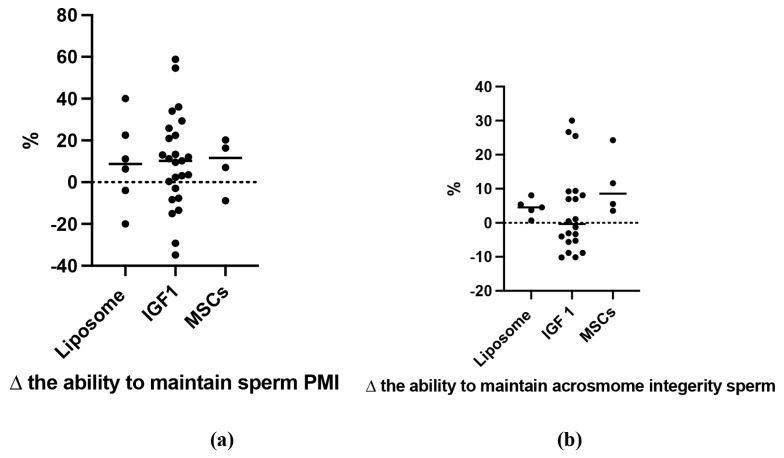
Percentage increase and decrease in the capability of growth factor in maintaining sperm (a) PMI and (b) Acrosome integerity compared to control. IGF-1, insulin growth factor-1; MSCs, mesenchymal stem cells; PMI, plasma membrane integrity.

The addition of IGF-1, liposomes, and MSC-derived secretomes to cryopreservation media significantly improved the PMI, acrosome integrity, and DNA integrity in thawed sperm compared to the control group, with SMDs of 1.82±0.362, 1.22±0.351, and 8.71±2.51, respectively. A significant increase in PMI post-thawing was observed in ram (5.03±2.26), canine (5.23±1.83), bull (1.27±0.53), and buffalo (1.88±0.53) sperm after GF supplementation (Figure 6). However, in boars, GF supplementation led to a slight decrease in PMI (−0.21±0.39), though this difference was not statistically significant. MSCs and IGF-1 both significantly increased PMI values (7.86±3.48 and 1.67±0.42, respectively). Notably, no data were available for human, rat, or stallion sperm, nor for MVs or exosomes, contributing to some variability across studies (Figure 7). As shown in Figure 7, GF supplementation preserved acrosome integrity in canine sperm (SMD: 4.55±1.47). However, the positive SMD values for acrosome integrity did not reach statistical significance in ram, bull, or buffalo sperm. MSC-derived secretomes, IGF-1, and liposomes showed higher effects on acrosome integrity (SMD values of 6.2±2.61 and 2.29± 0.83, respectively) compared to the control. Figure 8 illustrates the analysis of these GFs, with IGF-1 showing the highest percentage of acrosome integrity maintenance. Interestingly, despite yielding positive results, liposomes and MSC-derived secretomes did not significantly impact DNA integrity in post-thawing sperm from buffalo, bull, canine, or stallion (Figure 7). However, liposomes were notably effective in maintaining DNA integrity (SMD: 7.7±2.53), contributing to the considerable heterogeneity observed among studies evaluating DNA and acrosome integrity (Figure 8).

The addition of GFs to cryopreservation extender was found to significantly increase HOST grades in post-thawed sperm compared to the control group (SMD 2.02±1.12), showing significant heterogeneity (Table 1). Species-specific analysis revealed a significant improvement in HOST grades for bull and ram (SMD 2.75±0.16 and SMD 1.56±0.85, respectively), while no significant increase was observed in post-thawed canine sperm. Among the three GFs studied, only IGF-1 was found to significantly increase HOST grades compared to the control group (SMD 2.24±1.37). Studies investigating the impact of GF supplementation on MDA levels focused solely on IGF-1, with six different doses tested in frozen ram sperm. The findings indicated a significant association between IGF-1 supplementation and increased MDA levels in post-thawed ram sperm (SMD 6.3±6.26), accompanied by high heterogeneity (Table 2). The analysis revealed significant differences (p<0.05) in the provision of general GFs to animals compared to the control group. The effect size (SMD) on amplitude of lateral displacement (ALH) (0.61±0.289) and rapid motility (RM) values (1.48±0.718) showed significant differences. In contrast, progressive motility (pM) values (0.98±0.281) exhibited very significant differences (p<0.001), specifically in buffalo at 0.8±0.263 and cattle at 0.95±0.478 (Table 3). Average path velocity (VAP) values also significantly improved after the addition of GFs, reaching 1.93±0.385, with the most notable differences observed in buffalo at 0.91±0.36. Similar patterns were observed in velocity of curvilinear line (VCL) values (2.94±0.547) and velocity of straight line (VSL) values (2.52±0.482), particularly in cattle at 1.9±0.704. However, adverse effects were noted in animals after the addition of GFs (p<0.05) in the SMD value of LIN (−0.24±0.259), specifically in buffalo at −0.96±0.411. All categories with significant results were classified as having a large effect size due to the addition of these GFs. In contrast, animal-related factors were found to have a medium to low effect size. Spermatozoa kinematics were not found to be significantly different (p> 0.05) after the addition of GFs, with the BCF SMD value (−0.11±0.221) and the STR SMD value (−0.13±0.184) showing no significant changes.

## DISCUSSION

General growth factor effect as cryopreservation agent in quality post thawed mammal semen

In order to preserve the viability of sperms, cells, tissues, or organs, cryopreservation entails storing it at low temperatures. GFs, vital proteins that regulate spermatozoa processes and have a major influence on cryopreservation outcomes, are a crucial part of this process. The discovery that MSCs-secreted MVs are essential for repairing damaged sperm highlights the function of general GFs as cryopreservation agents in this study. The GFs, cytokines, and extracellular vesicles released by MSCs—multipotent stem cells originating from various tissues—promote cell proliferation and improve regenerative responses in mammalian sperm. Using liposomes, which are synthetic nanovesicles with a single lipid bilayer, is another development in cryopreservation. By using ultrasound to disrupt cell membranes, liposomes improve sperm cryopreservation by enabling the targeted delivery of medications and nutrients to tissues [18]. It has been demonstrated that incorporating antioxidants such as lycopene and quercetin greatly enhances sperm motility, mitochondrial activity, PMI, and viability [19]. Additionally, lecithin and other lipid components loaded into liposomes improve the efficiency of plasma membrane repair in ram spermatozoa throughout the freeze-thaw cycle [20]. The results of this study align with the success of liposomes as cryoprotective additives in a variety of animal species, such as horses [21], buffalo [22], sheep [23], pigs, and cattle [24], which have shown increased fertility after artificial insemination (AI). During the freeze-thaw process, the phospholipids and fatty acids in liposomes fuse with the sperm plasma membrane, possibly minimizing damage [25].

Exosomes, which are derived through the exocytosis pathway, carry a variety of cell secretory products in their cargo, such as proteins, miRNA, and mRNA. These bioactive compounds induce epigenetic and phenotypic changes in sperm, affecting viability, resistance to environmental factors, and regenerative capacity [14]. Exosomes contribute to sperm maturation, capacitation, acrosome reaction, and fertilization [15]. Ongoing research is investigating their potential to improve sperm freezing outcomes, including their associated GFs involved in tissue repair and healing [26]. IGF-1 regulates the cell cycle, promotes cell proliferation and prevents apoptosis [27,28]. IGF-1 in seminal plasma is also essential for spermatogenesis and testicular steroid production. Studies have indicated that IGF-1 supplementation improves sperm motility in various animals, including buffalo, sheep, yaks, stallions, and boars, while also increasing mitochondrial function [11,12,29]. These pathways promote cell proliferation, differentiation, and apoptosis, all of which are necessary for physiological processes, including spermatogenesis and steroidogenesis. This meta-analysis addresses a critical research gap by exploring the use of GFs in sperm extenders, which traditionally focus on antioxidant properties. The study highlights the underutilized potential of GFs, such as those found in exosomes and MSCs, which possess cell repair capabilities. These factors are particularly relevant for addressing sperm damage post-cryopreservation. The findings demonstrate that GF agents with repair properties, like those from MSCs, significantly enhance post-thaw sperm quality compared to traditional antioxidant-focused extenders, such as liposomes.

### Post-thawing motility in mammalian semen administered growth factors diluent

This study emphasizes the need to include GFs in sperm diluents for cryopreservation, since they generally improve post-thaw sperm motility in a variety of mammalian species. However, the data revealed an exception in boars (SMD −0.111) which in align with previous study [30]. The poor post-thaw semen quality in boars was attributed to the high concentration of unsaturated phospholipids and low plasma cholesterol content, which compromises sperm cell membrane function. Boar sperm are more vulnerable to membrane rupture during freezing, which results in decreased motility after thawing [31]. In all mammal except boar, GFs help mitigate the damage of unsaturated phospholipids by co-regulating protein phospholipid polarization and increasing energy metabolism in sperm after thawing consequently sustaining the sperm motility rate post-thaw [32]. Specifically, IGF-1 is shown to enhance sperm motility, likely by increasing carbohydrate metabolism, which in turn boosts energy production and supports motility [31].

In addition to IGF-1, other GFs, such as MSCs, MVs, and exosomes, participate in cell communication and post-sperm cell injury repair via intercellular paracrine pathways [33]. These pathways are essential for modulating the immunological response, such as increasing anti-apoptotic activity in sperm cells. These GFs promote sperm motility and protect against oxidative stress by minimizing physical damage [34]. Notably, these GFs have antioxidant action, neutralizing reactive oxygen species (ROS) [35] and enhance mitochondrial function via the Akt-1 pathway [36], which aids motility post-thaw.

Cryopreservation, however, is associated with sperm damage, particularly to the plasma membrane and oxidative stress. Recent advances in the use of manufactured and natural nanovesicles, such as liposomes and exosomes, have demonstrated the ability to regenerate and repair sperm damage induced by freeze-thaw cycles. Exosomes, in particular, have a pleiotropic effect by transporting antioxidants, lipids, and other bioactive compounds that control and aid sperm repair [12]. The data on liposomes in this investigation yielded conflicting findings. Liposomes help to preserve the plasma membrane’s cryo-stability, transfer plasma lipids and cholesterol, and cause membrane rearrangement after thawing [37]. They also help to repair sperm cells and improve GF function by addressing sperm plasma membrane damage [38]. However, liposomes bring additional obstacles, such as greater viscosity compared to other growth agents. Higher viscosity can sometimes have a deleterious impact on sperm motility in particular samples, as evidenced by some of the findings of this study [39].

### Post-thawing viability in mammalian semen administered growth factors diluent

Adding GFs to cryoprotectant diluents for sperm freezing has been proven to increase sperm quality measures [9,40]. These factors act as antioxidants, promoting cellular growth and differentiation [41]. Figure 2 shows that when human sperm are frozen in GF-enriched fluid, they outperform animal sperm in terms of vitality. This increase is due to GFs’ multiple activities in cell proliferation and differentiation [9]. GF addition in freezing solutions protects sperm during cryopreservation and improves vitality after post-thawing [9,42]. However, swine sperm exhibit worse viability when subjected to freezing conditions enriched with GFs, which can be linked to its increased vulnerability to oxidative stress, low temperatures, and cryoprotectant toxicity [43]. Increased amounts of polyunsaturated fatty acids produce oxidative stress, which compromises sperm function and structure. Low temperatures cause cold shock, structural damage, and reduced viability of post-thawed sperm [44]. GFs play an important role in controlling cellular activities such as growth stimulation and cell proliferation, which are required to maintain sperm viability following thawing [45]. By secreting certain proteins that regulate the immune response, promote regeneration, inhibit apoptosis, and lessen cellular scarring, MSCs have notably demonstrated promise in repairing processes [14]. The comprehensive use of GFs, including exosomes, MVs, MSCs, IGF-1, and liposomes, has been explored [45–47]. Each has distinct characteristics: liposomes act as additive cryoprotectants, MSCs aid in testicular development and spermatogenesis, IGF-1 promotes sperm maturation and quality, and exosomes and MVs increase sperm survival [37,47–49].

### Post-thawing plasma membrane integrity, acrosome and DNA integrity in mammalian semen administered growth factors diluent

PMI is the strength and resilience of the cell membrane in spermatozoa. Preserving PMI is critical because spermatozoa travel long distances, which is necessary for traversing the intricate path to successful egg fertilization [50]. If the cell membrane degrades or is damaged, spermatozoa may fail to fertilize the egg. Additionally, throughout the freezing and thawing process, PMI shields sperm against cold shock [51]. The results of this work show that PMI in spermatozoa from different mammalian species (ram, canine, bull, and buffalo) may be well preserved by combining GFs such as IGF-1, liposomes, and MSCs in cryopreservation solution.Notably, the addition of canine GFs resulted in the greatest increase in PMI compared to the other species. However, research on the utilization of GFs for canine sperm cryopreservation is sparse, and the materials used in this work came mostly from bull research. Previous studies have shown that MSC derivatives possess regenerative properties that help preserve sperm PMI [52]. These properties include the high expression of CD29, CD44, and CD90 surface markers, along with GFs, anti-apoptotic, and antioxidant properties (such as apolipoprotein E and thioredoxin). These elements work together in various intracellular communication processes to maintain sperm ultrastructure or repair the plasma membrane, thus protecting it from adverse conditions [53]. Furthermore, MSCs secrete critical extracellular proteins, such as annexin, dysferlin, and fibronectin, which may contribute to maintaining PMI in spermatozoa [14]. The GFs secreted by MSCs and IGF-1 can support cell viability and prevent apoptosis through the SFRP2 pathway in the nucleus [54].

Both acrosome and DNA integrity are essential for successful fertilization and normal embryo development. Damage to acrosomes and DNA in sperm can arise from various factors, including the freezing-thawing process [55]. The addition of GFs significantly preserved acrosome integrity in canine spermatozoa after thawing compared to the other mammalian species studied. Although a positive effect was observed in other species (ram, bull, and buffalo), the results were not statistically significant. The analysis of each GF revealed that MSCs and liposomes can keep acrosomes intact during sperm cryopreservation. Furthermore, only liposomes significantly retained DNA integrity as compared to MSCs. Liposomes form a protective barrier surrounding sperm cells, aiding in cell integrity and preventing damage from high-temperature swings during cryopreservation [24].

### Post-thawing on hyper-osmotic swelling test and malondialdehid values in mammalian semen administered growth factors diluent

HOST assesses sperm’s response to osmotic pressure changes, which is crucial for identifying viable and functionally competent sperm. The test involves exposing sperm to the hypoosmotic solution, where live sperms respond by taking up water, causing tail swelling or curling [56]. GFs supplementation safeguards membrane integrity during cryopreservation against extreme osmotic changes. IGF-1, present in seminal plasma, acts as an antioxidant [11,57]. A deficiency in IGF-1 may lead to mitochondrial dysfunction and oxidative stress [58]. Among the 16 studies reviewed, only liposomes and MSCs were tested for HOST on post-thawed canine sperm. Despite their antioxidant effects, their impact on membrane integrity was insignificant, indicating potential species-specific responses [12]. Factors such as cryo-sensitivity, adaptability to osmotic changes, and AQP channels may contribute to these variations among species. Notably, only IGF-1 showed a significant improvement in HOST grades post-thaw, suggesting the unique receptivity of sperm from the studied species to IGF-1. Further exploration of molecular pathways and receptor interactions involved in GF effects could provide insights into these observed outcomes.

The observed elevation in MDA levels associated with IGF-1 supplementation in post-thawed ram sperm suggests a potential induction of oxidative stress or lipid peroxidation. However, the diverse responses across different post-thaw sperm quality parameters to IGF-1 indicate a complex relationship, potentially influenced by various biological factors or varying IGF-1 concentrations. These findings emphasize the need for further investigations to elucidate the mechanisms contributing to the increased MDA levels following IGF-1 supplementation. Additionally, exploring the dose-dependent effects IGF-1 on oxidative stress markers in frozen sperm could provide valuable insights into its potential role in oxidative damage pathways and sperm viability post-thaw.

### Post-thawing on sperm kinematics using computer-assisted semen analysis

CASA which quantifies the kinematic properties of spermatozoa, is essential for assessing sperm quality. In these studies, the CASA parameters were tailored to the specific semen samples under investigation. Across species, GFs have been shown to positively influence sperm kinematics, likely due to their metabolic regulatory functions via corresponding receptors [10]. Oxidative stress is exacerbated by semen dilution, and cryopreservation procedures further increase the production of ROS from mitochondrial activity (mROS), which negatively affects the kinematic quality of thawed sperm [59]. GFs that enhance ATP metabolism and modulate mitochondrial superoxide production, such as IGF-1 and MSCs, have protective effects [13,60]. IGF-1 reduces oxidative stress by promoting mitochondrial activity, preventing cellular damage, and activating anti-apoptotic pathways [58,61]. Glycolytic enzymes and mitochondria are critical energy sources for sperm motility, which depends on ATP synthesis [62]. The reversible attachment of liposomes during cryopreservation improves sperm movement properties by reducing mitochondrial damage [53]. Increased CASA parameters, particularly those associated with IGF-1, result from the maintenance of mitochondrial function, protection against malfunction, and antioxidant benefits [58]. Parameters such as VAP, VCL, VSL, and VAP are key metrics positively correlated with fertility and reproductive potential [63,64]. GFs do not interfere with the linear forward propulsion of hyperactive spermatozoa [65]. Additionally, GFs do not affect parameters related to linear and forward movement, such as STR and LIN values. Although GFs do not have a discernible effect on BCF, a measure of sperm strength, BCF is predictive of in vitro fertilization outcomes [31]. Sperm functional tests, such as linear movement and hyperactivity, are valuable indicators of semen quality post-freezing and thawing.

This meta-analysis conclusively demonstrates that GFs can improve post-thaw sperm quality by elevating hyperactivity without negatively impacting sperm motility or linear progression. The processes underlying these enhancements are likely due to the availability of repair agents and antioxidants that minimize cellular damage during cryopreservation. Further meta-analyses examining additional supplementary additives could enhance our comprehension of sperm preservation by elucidating the various factors affecting sperm viability and motility post-thaw, thereby optimizing cryopreservation protocols and improving reproductive outcomes.

## CONCLUSION

In conclusion, the effectiveness of GFs on sperm quality is dependent on the specific type of factor and the sperm source. This meta-analysis reveals that while lysosomes and IGF-1 are frequently utilized, only MSCs and exosomes consistently improve all sperm quality parameters. MSCs and exosomes show significant promise in repairing and recovering sperm cells after thawing, particularly in cryopreservation, where sperm viability is often compromised. In contrast, liposomes and IGF-s yield inconsistent results; liposomes can enhance viability but may cause oxidative stress, while IGF-s can improve motility but may damage DNA at elevated concentrations. Comprehensive studies are needed to further evaluate the potential of GFs like MSCs and exosomes as effective agents in post-cryopreservation applications.

## Figures and Tables

**Table 1:** Compilation of the research works incorporated in the meta-analysis

No	Animals	Growth factors	Extender medium	Dosage	Control number	Treatment number	References
1	Buffallo	Liposome	TEYG	0–0.25 mL	5	5	[53]
2	Buffallo	Liposome	TEYG	0–0.25 mL	50	50	[22]
3	Buffallo	IGF-1	TEYG	0–350 ng/mL	4	12	[22]
4	Buffallo	IGF-1	TEYG	0–150 ng/mL	20	60	[11]
5	Bull	IGF-1	TEYG	0–100 ng/mL	24	24	[66]
6	Bull	Liposome	TEYG	0–0.25 mL	3	3	[67]
7	Bull	IGF-1	TEYG	0–450 ng/mL	7	63	[68]
8	Bull	IGF-1	TEYG	0–200 ng/mL	15	48	[69]
9	Bull	IGF-1	Tris, TEYG, ASL	0–150 ng/mL	32	96	[70]
10	Bull	Liposome	Tris, TEYG, ASL; Optixell®	0–0.25 mL	12	24	[70]
11	Bull	Liposome	TEY; Optixell®	0–0.25 mL	5	5	[71]
12	Canine	Liposome	TEY	6%	7	7	[39]
13	Canine	MSCs	Tris-G	0%–15%	40	60	[52]
14	Canine	MSCs	Tris-G	0–50 ug/mL	16	16	[14]
15	Canine	MSCs	Tris-G	0–5 mM	4	8	[72]
16	Ram	IGF-1	TEYG	0–300 ng/mL	12	64	[73]
17	Ram	IGF 1	SMEYG	0–24 ng/mL	12	24	[74]
18	Ram	IGF-1	Tris-G	0–259 ng/mL	25	75	[75]
19	Mouse	Microvesicle	TEYG	0–100 ug/mL	24	72	[46]
20	Human	Exosome	Ham 10 medium	0–2 mg	25	75	[76]
21	Human	Microvesicle	Ham 10 medium	0–2 mg	25	75	[76]
22	Pig	IGF-1	LEY	0–30 ng/mL	8	16	[31]
23	Pig	Liposome	Androstar®	3 mM	15	15	[35]
24	Stalions	Liposome	SMEYG	0–7 mg	5	5	[77]
25	Stalions	Liposome	INRA 96®	0–50 uL	30	30	[21]

TEYG, tris egg yolk-glycerol; IGF-1, insulin growth factor-1; ASL, Andromed Soya-Lecithin; TEY, tris egg yolk; MSCs, mesenchymal stem cells; SMEYG, skim milk egg yolk glycerol; Tris-G, tris-glycerol; LEY, lactose egg yolk.

**Table 2:** General sperm quality on growth factors administration

Parameter	Subgroup	n	Effect size				

			SMD (95% CI)	SE	p-value	I^2^ (%)	p-value
Motility	General	66	2.56 [1.97 to 3.15]	0.303	<0.001	95.340	<0.001
Viability	General	38	3.53 [2.7 to 4.35]	0.423	<0.001	96.060	<0.001
Acrosome integrity	General	29	1.22 [0.53 to 1.91]	0.351	0.001	93.970	<0.001
PMI	General	35	1.82 [1.11 to 2.53]	0.362	<0.001	94.870	<0.001
DNA integrity	General	6	8.73 [3.8 to 13.65]	2.514	0.001	98.110	<0.001
HOST	General	16	2.02 [1.12 to 2.93]	0.462	0.000	94.670	<0.001
MDA	General	6	6.3 [0.76 to 11.84]	2.827	0.026	97.220	<0.001
CASA							
ALH	General	33	0.61 [0.04 to 1.18]	0.289	0.035	90.840	<0.001
BCF	General	25	−0.11 [−0.54 to 0.32]	0.221	0.623	86.850	0.000
LIN	General	40	−0.24 [−0.74 to 0.27]	0.259	0.359	91.930	<0.001
PM	General	42	0.98 [0.43 to 1.53]	0.281	0.000	92.950	<0.001
RM	General	11	1.48 [0.07 to 2.88]	0.718	0.040	86.380	0.000
STR	General	34	−0.13 [−0.49 to 0.23]	0.184	0.476	79.260	0.000
VAP	General	38	1.93 [1.17 to 2.68]	0.385	<0.001	95.170	<0.001
VCL	General	34	2.94 [1.87 to 4.01]	0.547	<0.001	96.320	<0.001
VSL	General	35	2.52 [1.57 to 3.46]	0.482	<0.001	95.580	<0.001

SMD, standardized mean differences; CI, confidence interval; SE, standard error; PMI, plasma membrane integrity; HOST, hyper-osmotic swelling test; MDA, malondialdehid; CASA, computer-assisted semen analysis; ALH, amplitude of lateral displacement; BCF, beat cross frequency; LIN, linearity; PM, progressive motility; RM, rapidity of movement; STR, straightness; VAP, velocity of average path; VCL, velocity of curvilinear line; VSL, velocity of straight line.

**Table 3:** Sperm kinematics on growth factor administration in each mammals species

Parameter	Subgroup	n	Effect size				

			SMD (95% CI)	SE	p-value	I^2^ (%)	p-value
ALH	General animals	33	0.61	0.289	0.035	90.840	<0.001
	Buffallo	4	2.2	0.296	<0.001	86.270	<0.001
	Bull	21	0.02	0.347	0.950	90.930	<0.001
	Canine	8	1.21	0.534	0.023	63.560	0.041
	Growth factor						
	IGF-1	20	0.16	0.304	0.601	81.510	<0.001
	Liposome	6	1.69	1.036	0.102	97.130	<0.001
	MSCs	7	1.04	0.559	0.064	91.280	<0.001
BCF	General animals	25	−0.11	0.221	0.623	86.850	0.000
	Buffallo	4	0.78	0.414	0.059	73.490	<0.001
	Bull	21	−0.34	0.247	0.163	79.870	<0.001
	Growth factor						
	IGF-1	20	−0.13	0.269	0.622	77.360	<0.001
	Liposome	5	−0.09	0.455	0.835	87.850	<0.001
LIN	General animals	40	−0.24	0.259	0.359	91.930	<0.001
	Boar	2	0.29	0.356	0.423	0.000	0.580
	Buffallo	4	−0.96	0.411	0.020	86.540	0.000
	Bull	21	0.21	0.254	0.407	72.900	<0.001
	Canine	7	1.03	0.300	0.001	71.550	0.002
	Human	6	−3.43	1.083	0.002	97.560	<0.001
	Growth factor						
	Exosome	3	−6.39	1.530	0.000	93.730	<0.001
	IGF-1	22	0.05	0.143	0.736	33.690	0.063
	Liposome	5	−0.4	0.744	0.592	91.990	<0.001
	Microvesicle	3	−0.65	0.862	0.451	95.580	<0.001
	MSCs	7	1.03	0.300	0.001	71.550	0.002
PM	General animals	42	0.98	0.281	0.000	92.950	<0.001
	Boar	2	−0.15	0.356	0.673	0.000	0.423
	Buffallo	7	0.80	0.263	0.002	64.830	0.009
	Bull	25	0.95	0.478	0.048	93.530	<0.001
	Canine	2	0.36	0.581	0.532	70.990	0.063
	Human	6	1.96	0.883	0.026	97.170	<0.001
	Growth factor						
	Exosome	3	2.83	1.198	0.018	96.150	<0.001
	IGF-1	26	0.65	0.375	0.084	91.580	<0.001
	Liposome	7	1.41	0.559	0.012	91.120	<0.001
	Microvesicle	3	1.11	1.317	0.401	97.620	<0.001
	MSCs	1	0.87	0.331	NA	NA	NA
RM	General animals	11	1.48	0.718	0.040	86.380	0.000
	Buffallo	4	1.48	0.833	0.076	93.410	<0.001
	Bull	7	1.52	1.187	0.201	91.770	<0.001
	Growth factor						
	IGF-1	10	1.32	0.818	0.107	90.160	<0.001
	Liposome	1	2.96	0.290	NA	NA	NA
STR	General animals	34	−0.13	0.184	0.476	79.260	0.000
	Boar	2	0.02	0.354	0.947	0.000	0.634
	Buffallo	4	−0.78	0.221	0.000	55.650	0.080
	Bull	21	0.11	0.212	0.597	62.420	0.080
	Canine	7	−0.1	0.577	0.867	91.110	<0.001
	Growth factor						
	IGF-1	22	−0.15	0.115	0.180	6.360	0.375
	Liposome	5	0.25	0.765	0.741	93.290	<0.001
	MSCs	7	−0.1	0.577	0.867	91.110	<0.001
VAP	General animals	38	1.93	0.385	<0.001	95.170	<0.001
	Buffallo	4	0.91	0.369	0.013	83.400	0.000
	Bull	22	0.9	0.496	0.069	91.780	<0.001
	Canine	5	0.62	0.490	0.206	88.590	<0.001
	Human	6	7.56	0.817	<0.001	84.590	<0.001
	Stalion	1	0	0.632	NA	NA	NA
	Growth factor						
	Exosome	3	7.84	1.194	<0.001	83.910	0.002
	IGF-1	20	0.67	0.356	0.060	86.200	<0.001
	Liposome	8	0.65	0.867	0.455	95.570	<0.001
	Microvesicle	3	7.36	1.362	<0.001	89.200	0.000
	MSCs	4	0.62	0.583	0.284	91.410	<0.001
VCL	General animals	34	2.94	0.547	<0.001	96.320	<0.001
	Buffallo	1	0.97	0.211	NA	NA	NA
	Bull	22	1.39	0.729	0.056	94.420	<0.001
	Canine	5	0.52	0.346	0.135	78.170	0.001
	Human	6	11.55	1.354	<0.001	88.280	<0.001
	Growth factor						
	Exosome	3	10.04	1.644	<0.001	86.730	0.001
	IGF-1	17	1.31	0.630	0.037	89.960	<0.001
	Liposome	7	0.98	1.366	0.475	97.350	<0.001
	Microvesicle	3	13.26	2.447	<0.001	90.080	0.000
	MSCs	4	0.58	0.407	0.154	83.400	0.000
VSL	General animals	35	2.52	0.482	<0.001	95.580	<0.001
	Buffallo	1	0.53	0.203	NA	NA	
	Bull	22	1.9	0.704	0.007	93.790	<0.001
	Canine	5	0.54	0.522	0.298	89.870	<0.001
	Human	6	7.13	0.789	<0.001	85.090	<0.001
	Stalion	1	−0.06	0.633	NA	NA	NA
VCL	Growth factor						
	Exosome	3	7.22	1.221	<0.001	86.750	0.001
	IGF-1	17	2.28	2.277	0.776	3.779	0.766
	Liposome	8	0.69	0.690	−0.978	2.357	0.851
	Microvesicle	3	7.13	7.131	4.549	9.714	1.317
	MSCs	4	0.32	0.321	−0.806	1.448	0.575

SMD, standardized mean differences; CI, confidence interval; SE, standard error; ALH, amplitude of lateral displacement; MSCs, mesenchymal stem cells; IGF-1, insulin growth factor-1; BCF, beat cross frequency; LIN, linearity; PM, progressive motility; RM, rapidity of movement; STR, straightness; VAP, velocity of average path; VCL, velocity of curvilinear line; VSL, velocity of straight line.
